# Case report: POEMS syndrome with portal hypertension

**DOI:** 10.3389/fmed.2024.1373397

**Published:** 2024-07-23

**Authors:** Xiaotong Xu, Changyou Jing, Tong Zhu, Minjie Jiang, Yunlai Fu, Fang Xie, Jianjun Li, Qinghua Meng

**Affiliations:** ^1^Beijing Institute of Hepatology, Beijing Youan Hospital, Capital Medical University, Beijing, China; ^2^Department of Oncology, Beijing Youan Hospital, Capital Medical University, Beijing, China; ^3^Interventional Therapy Center for Oncology, Beijing Youan Hospital, Capital Medical University, Beijing, China

**Keywords:** POEMS syndrome, portal hypertension, case report, ascites, hydrothorax

## Abstract

This patient was an elderly patient with abdominal distension and shortness of breath. According to relevant examinations, his condition was initially considered to be related to cirrhosis, but pathological biopsy confirmed the diagnosis of noncirrhotic portal hypertension of unknown etiology. The portal vein pressure was significantly reduced after transjugular intrahepatic portosystemic shunt (TIPS). Nevertheless, the relief of the hydrothorax and ascites was not significant, and the numbness in both lower limbs gradually worsened. POEMS syndrome was ultimately diagnosed following a comprehensive examination. After two courses of bortezomib combined with dexamethasone, the patient died due to a systemic infection. The clinical symptoms of the patient were atypical, as was the presence of portal hypertension, which hindered the diagnosis of POEMS. Due to the patient’s advanced age, the diagnosis was delayed, and the prognosis was poor. This case reminds clinicians that POEMS patients can also have portal hypertension as the main manifestation.

## 1 Introduction

POEMS syndrome, also known as Crow Fukase syndrome, was proposed by Bardwick in 1980 (1). It is a rare multisystem disease caused by plasma cell proliferation. The clinical manifestations of POEMS mainly include peripheral neuropathy, organomegaly, endocrinopathy, M protein elevation, and skin changes (2, 3). Patients rarely present with hydrothorax and ascites as the main clinical manifestations. Therefore, these patients may be misdiagnosed or their diagnosis may be missed. We reported a case of a POEMS patient with portal hypertension as the main clinical manifestation.

## 2 Case presentation

The patient, a 70-year-old male, was diagnosed with liver cirrhosis 11 months prior, and he had been experiencing abdominal distension and shortness of breath for more than 1 month. He visited our hospital in June 2023. The patient developed abdominal distension without an obvious cause 11 months prior and was diagnosed with liver cirrhosis at a local hospital. Albumin supplementation and diuretic treatment alleviated symptoms to some extent. One-month prior, the patient experienced abdominal distension accompanied by shortness of breath. After admission to the local hospital for albumin supplementation, diuresis, and paracentesis, his condition improved. However, the discomfort described above persisted. To further clarify the diagnosis and treatment, the patient sought medical attention at our hospital. During the disease course, the patient experienced a poor appetite, poor sleep, normal defecation, and decreased urination.

He had been taking oral tablets to control hypertension. Renal dysfunction was discovered 3 months prior. He denied a history of alcohol use. Physical examination revealed the following: a height of 176 cm, a weight of 64 kg, a body temperature of 36.3°C, a pulse of 62 beats/min, an exhalation of 19 beaths/min, and a blood pressure of 147/84 mmHg. The typical signs of liver cirrhosis, including spider haemangioma, engorged paraumbilical veins, or palm erythema, were all negative. Cognitive function appeared normal, and cyanosis of the lips, jaundice of the skin or sclera, and palpable swelling of superficial lymph nodes were all absent. He presented with coarse breathing sounds in both lungs and weak vocal resonance in the right lung. His heart rhythm was consistent, with no additional heartbeats, murmurs, or pericardial friction sounds. The abdomen was full, without tenderness, rebound pain, or muscle tension. The liver and spleen were not palpable, but shifting dullness was detected. Both lower limbs were free of oedema. The diagnostic examination results are shown in Table 1.

**Table 1:** Diagnostic examination during hospitalization.

Routine examination (evidence supporting portal hypertension)					
Blood routine	WBC 3.82*10^9^/L, HGB 92 g/L(130–175)↓, PLT 113*10^9^/L↓(125–350)				
Liver function (before TIPS)	ALT 5 U/L, AST 9 U/L, γ-GT 18 U/L, ALP 74 U/L,TBIL 15.8 μmol/L, ALB 26.4 g/L↓(40–55), CHE 2259 U/L↓(4,000–13,000),GLB 22 g/L(20–40), ALB/GLB 1.2(1.2–1.4)				
Liver function (after TIPS)	ALT 4 U/L, AST 8 U/L, γ-GT 12 U/L, ALP 57 U/L,TBIL 13.9 μmol/L, ALB 29 g/L↓(40–55), CHE 2277 U/L↓(4,000–13,000),GLB 24.2 g/L(20–40), ALB/GLB 1.2(1.2–1.4)				
Renal function	Cr 144 μmol/L↑(57–111), eGFR 42.1 mL/min↓			NH3	Normal
Coagulation	PTA 78.7%(74.4–120%), IR 1.08(0.91–1.15),PT 9.6(7.67–10.5),APTT 49.9↑(26.6–43.6)				
Immunoglobulin	IgA 5.97 g/L↑(1.0–4.2), IgG,IgM(normal), Complement C3 0.497 g/L(0.7–1.4), Complement C4(normal)				
Infection markers	Normal	Autoantibody Series	Normal	Liver antigenic spectrum	Normal
Hydrothorax (before TIPS)	Light yellow transparent. Total protein 22.6 g/L, albumin 13.3 g/L, total cell count 71 * 10^6^/L, Rivalta test (−).Culture of *Staphylococcus epidermidis* and Fermented Corynebacterium in hydrothorax				
Hydrothorax (after TIPS)	Light yellow slightly gray. Total albumin 25.9 g/L, albumin 22.6 g/L, cell count 1,083 * 10^9^/L, Rivalta test (+). No pathogenic bacteria were found in the hydrothorax				
Serum-pleural effusion albumin gap (before TIPS)		13.1 g/L	Serum-pleural effusion albumin gap (after TIPS)		6.4 g/L
Ascites (before TIPS)	Light yellow transparent Total protein 16.2 g/L, albumin 8.8 g/L, cell total 105 * 10^6^/L, Rivalta test (−) No pathogenic bacteria were found in the ascites culture				
PC	43.6%↓(70–140%)		PS	55.3%↓(60–130%)	
ECG	Sinus rhythm, limb lead low voltage, prolonged QTc interval, abnormal electrocardiogram				
Echocardiography	Reduced left ventricular diastolic function, small pericardial effusion, small amount of mitral, tricuspid, and aortic regurgitation				
Liver stiffness (before TIPS)	38.8 kPa (F4)		Liver stiffness (after TIPS)		20.3 kPa (F4)
Pulmonary CT	There may be slight inflammation in both lungs, right hydrothorax, and passive atelectasis in the middle and lower lobes of the right lung				
Abdominal enhanced CT	Cirrhosis, splenomegaly, formation of collateral circulation, ascites. Cholecystitis, gallstones. Bilateral renal cysts. Mild dilation of the right renal pelvis and upper ureter. Increased density of the lumbar pyramidal body and nodular shadows in the thoracic and lumbar vertebrae				
Endoscopic examination	Portal hypertensive gastropathy and gastric xanthoma				
TIPS	The main portal vein diameter was 1.7 cm. The pressure of the inferior vena cava and right atrium was 5 mmHg and 4 mmHg, respectively, and the portal vein pressure was 23 mmHg. After stent implantation, the portal vein pressure was 16 mmHg, and the pressure in the inferior vena cava and right atrium was 10 mmHg and 8 mmHg, respectively.				
Transjugular liver biopsy pathology	Consistent with noncirrhotic portal hypertension. A total of 17 mildly dilated small and medium-sized portal areas were observed in the slices, with interstitial fibrosis and mild to moderate infiltration of mononuclear cells. Most of them did not show corresponding caliber portal veins, small arteries did not show any special features, small bile ducts were preserved, and the peripheral zone showed significant proliferation of fine bile ducts, accompanied by interfacial inflammation. The lobular structure was preserved, with mild inflammation and widespread perisinusoidal fibrosis, mainly in bands 3 and 2. CD34 immunostaining was positive for most of the liver sinusoid endothelium around the portal area, indicating that the advantage of arterial blood supply was consistent with portal hypertension. Immunohistochemistry of HBsAg (−), HBcAg (−), CK7 (bile duct+), CK19 (bile duct+), MUM1 (plasma cell+), CD34 (blood vessel+); Special staining: iron (Perls method, −), copper (Rhodanine method, −)				
Further examination (evidence supporting POEMS)					
Electromyography	Electrophysiological manifestations of multiple peripheral nerve damage (involving both motor and sensory nerves, axonal and myelin sheath damage)				
Bone marrow biopsy	Triple lineage enhanced life and bone marrow imaging, with a small amount of abnormal plasma cells visible				
Serum protein electrophoresis	Monoclonal immunoglobulin type (IgA- λ type)			VEGF	168.46 pg./mL↑(<160)
Serum-free light chain	Serum-free Kappa light chain 215.38 mg/L ↑ (3.3–19.4), serum-free lambda light chain 226.35 mg/L ↑ (5.71–26.30), serum-free Kappa light chain/serum-free lambda 0.9515 (0.26–1.65)				
Whole-body bone imaging	Bilateral multiple ribs, multiple pyramids in the spine (with neck 7 and waist 3 as the main focus), bilateral ilium, and active bone salt metabolism in the upper femur, combined with computed tomography fusion imaging and medical history, were consistent with bone changes in POEMS syndrome				
Sex hormone	Oestradiol 44.13 pg/mL ↑ (0–39.8), testosterone 0.12 ng/mL ↓ (0.28–1.22), follicle-stimulating hormone 0.02 IU/L ↓ (1.4–18.1), luteinizing hormone<0.1 IU/L ↓ (1.5–9.3), prolactin 20.1 ng/mL ↑ (2.1–17.7)				
Thyroid function testing	FT3 2 pg./mL↓(2.3–4.2), FT4 0.53 ng/dL↓(0.89–1.76), TSH 5.85 uIU/mL↑(0.55–4.78)				
Chromosome karyotype analysis	Analysis of 20 metaphase divisions revealed no clonal structural or numerical abnormalities				
Urinary protein electrophoresis	Normal	β2 Glycoprotein 1 antibody	Normal	Urine Bence-Jones protein electrophoresis	Normal
Anticardiolipin antibody	Normal	Urinary free light chain	Normal	Parathyroid hormone	Normal

WBC, white blood cell; HGB, red blood cell; PLT, platelet; ALT, alanine aminotransferase; AST, aspartate aminotransferase;γ-GT, glutamyl transpeptidase; ALP, alkaline phosphatase; ALB, albumin; TBIL, total bilirubin; DBIL, direct bilirubin; CHE, cholinesterase; GLB, globulin; Cr, serum creatinine; eGFR, estimated glomerular filtration rate; NH3, plasma ammonia; PTA, prothrombin activity; INR, international normalized ratio; PT, prothrombin time; APTT, activated partial thromboplastin time; IgA, immunoglobulin A; IgG, immunoglobulin G; IgM, immunoglobulin M; PC, protein C activity; PS, protein S activity; TIPS, transjugular intrahepatic portosystemic shunt; ECG, electrocardiogram; HBsAg, hepatitis B virus surface antigen; HBcAg, hepatitis B virus core antigen; CK7, cytokeratin 7; CK19, cytokeratin 19; MUM1,multiple myeloma oncogene 1; CD34, cluster of differentiation 34;VEGF, vascular endothelial growth factor; FT3, free triiodothyronine 3; FT4, free triiodothyronine 4; TSH, thyrotropin, thyroid stimulating hormone; CT, computed tomography.

The final diagnoses were noncirrhotic portal hypertension, splenic hyperfunction, hypoalbuminaemia, ascites, right hydrothorax, chronic renal insufficiency (chronic kidney disease, stage 3), and grade 3 hypertension (extremely high-risk group). The patient received symptomatic treatment including albumin supplementation, diuresis, improvement of renal perfusion, and thoracic and abdominal paracentesis. Given the refractory hydrothorax and ascites and the patient’s advanced age, a transjugular intrahepatic portosystemic shunt (TIPS) was applied, and a transjugular liver biopsy was performed to determine the cause of the condition. After TIPS placement, the patient was discharged without any discomfort.

**Figure 1:**
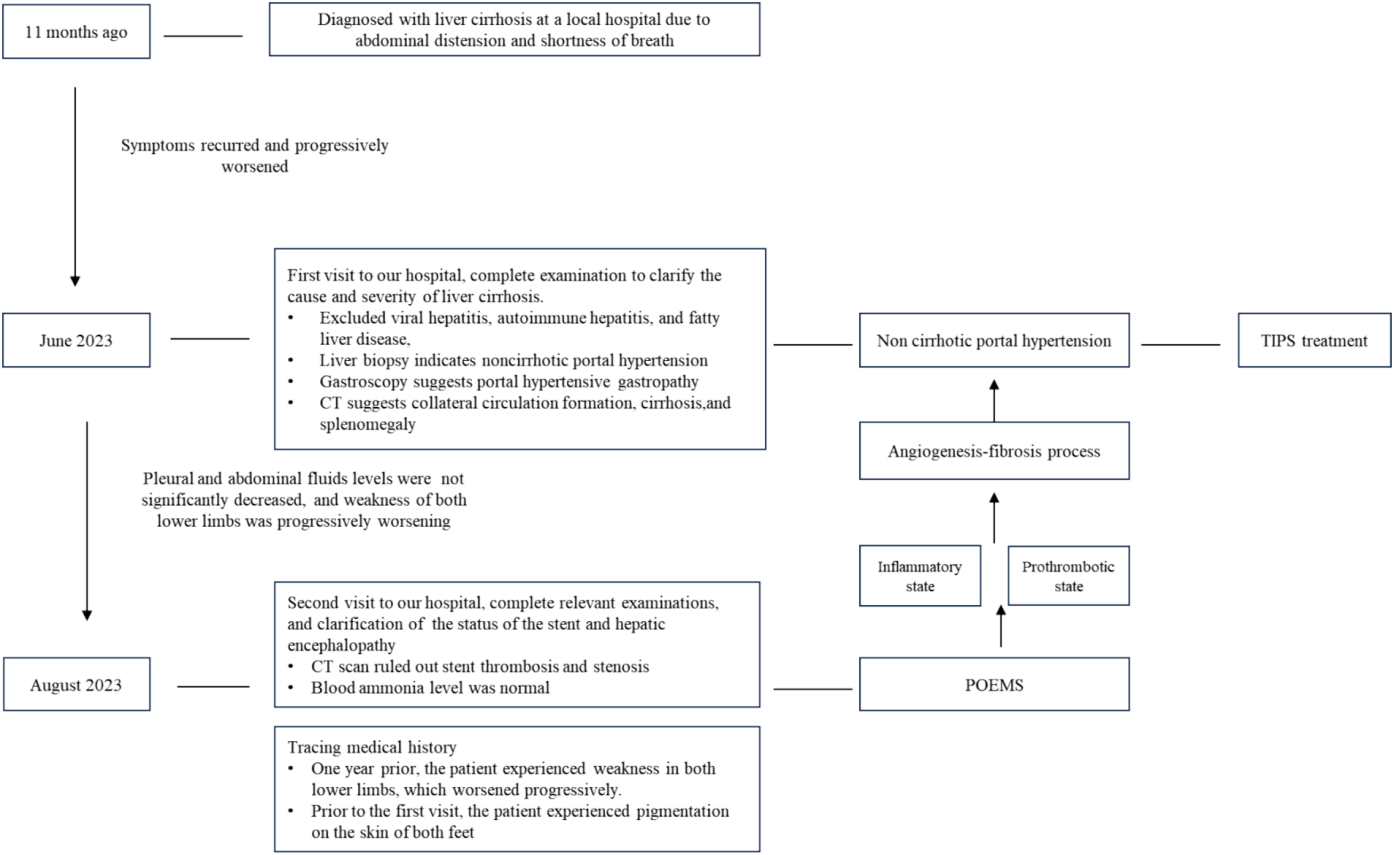
Diagnostic flow chart.

Two weeks after TIPS placement, the patient was admitted to the local hospital for symptomatic treatment due to pleural effusion and ascites. One and a half months after TIPS surgery, the patient was referred to our hospital again due to nausea, fatigue, abdominal distension, and shortness of breath. Re-examination via computed tomography showed no thrombosis or stenosis in the TIPS stent. After symptomatic treatment involving liver protection, diuresis, albumin supplementation, and fluid drainage, the patient’s condition improved slightly. Considering the persistent recurrent pleural and abdominal fluid accumulation after TIPS placement and concomitant, lower limb weakness but lack of hepatic encephalopathy, the patient’s medical history was traced again. One-year prior, the patient had developed weakness in both lower limbs, and in May of this year, melanin deposition appeared on the skin of both feet. One and a half months after TIPS placement, the weakness in both lower limbs gradually worsened, and hydrothorax and ascites recurred. However, the patient did not experience significant relief after reducing portal pressure. Based on the above symptoms, relevant examinations of the blood system improved, and the diagnosis of POEMS syndrome with portal hypertension was ultimately confirmed. Subsequently, bortezomib combined with glucocorticoids was administered for treatment. After two courses of treatment, the patient died due to pneumonia and infection. The diagnostic process is shown in Figure 1.

## 3 Discussion

The patient in this case was an elderly male with refractory hydrothorax and ascites. Combined with routine laboratory and imaging examinations, the initial diagnosis was consistent with the clinical characteristics of liver cirrhosis (2018) (4). Ascites is a common complication of liver cirrhosis, with an annual incidence rate of 5–10%. The 1-year and 5-year mortality rates for patients with cirrhosis and ascites are 30% and 70%, respectively, and the median survival time for patients with refractory ascites is only 6 months (5, 6). TIPS placement is one of the main methods used to reduce portal vein pressure by establishing a new channel between the portal vein and hepatic vein, which can quickly reduce portal vein pressure in patients with cirrhosis and without cirrhosis, achieve hemostasis, and relieve ascites (7–10). In addition, compared with the treatment of complications of portal hypertension in cirrhosis, TIPS placement is advantageous for treating complications of noncirrhotic portal hypertension as the latter has relatively good liver function with low incidences of liver failure, and hepatic encephalopathy (11). Compared to abdominal paracentesis, TIPS placement significantly improves the transplant-free survival rate of patients with refractory ascites and cirrhosis and reduces the risk of recurrent ascites and hepatorenal syndrome, but increases the risk of HE (12).

Viral infection and autoimmune liver disease were ruled out for this patient. To further clarify the cause of liver disease, the patient underwent liver biopsy. Due to the presence of ascites in the patient, a transjugular liver biopsy was performed (13). Transjugular liver biopsy has expanded the indications for liver biopsy, and research has shown that its effectiveness is comparable to that of percutaneous liver biopsy (14, 15). Finally, the patient was diagnosed with noncirrhotic portal hypertension. However, the patient’s symptoms of hydrothorax and ascites did not improve after TIPS. The common cause of persistent ascites after TIPS surgery is obstruction or stenosis of the TIPS. However subsequent CT scans showed no abnormalities, and lower limb symptoms worsened after surgery without significant abnormalities in blood ammonia levels or direction or computational ability.

To clarify the etiology of portal hypertension, a combination of patient history and further relevant examinations were needed (16). The patient had peripheral nerve damage, abnormal monoclonal plasma cell proliferation (IgA-λ), clerosing bone lesions, elevated VEGF, and abnormal thyroid function. Ultimately, POEMS syndrome diagnosed. Portal hypertension may be associated with POEMS.

Clinically, POEMS is characterized by peripheral polyneuropathy (P), organ enlargement (O), endocrine disorders (E), M proteinaemia (M), and skin changes (S). The pathogenesis may be related to the excessive secretion of M protein, inflammatory cytokines, and vascular endothelial growth factor. This secretion may result in the dysfunction of multiple systems, such as the nervous and endocrine systems. Usually, the disease begins in middle age and occurs between the ages of 40 and 60. It affects mainly males, with an incidence rate of 0.3/100000 (3, 17–19). Diagnosis needs to be based on a combination of mandatory criteria, primary criteria, and secondary criteria. When 2 mandatory criteria,1 primary criterion, and 1 secondary criterion are met, a clear diagnosis can be made (2).

The association between portal hypertension and POEMS syndrome has not yet been confirmed but may be related to vascular defects caused by inflammation and elevated levels of vascular endothelial growth factor (1, 20–23). The liver abnormalities reported in POEMS syndrome can be manifested as Budd Chiari syndrome, nonthrombotic hepatic venous obstruction, liver massess, hemangioma, ascites with hepatosplenomegaly, idiopathic cirrhosis, portal hypertension, and portal hypertension with neutrocytic ascites (24). Therfore clinicians should identify the etiology of liver disease upon initial diagnosis, and timely treatment targeted at the underlying disease.

The most common cause of portal hypertension is cirrhosis (accounting for more than 90% cases), but approximately 10% of portal hypertension cases are not caused by cirrhosis; this condition is known as noncirrhotic portal hypertension. Noncirrhotic portal hypertension (NCPH) (16, 25, 26) includes noncirrhotic portal fibrosis (NCPF), idiopathic portal hypertension (IPH), and extrahepatic portal venous obstruction (EHPVO). It is a disease characterized by portal hypertension (PHT), the preservation of liver synthesis function, and normal or mild elevation of the hepatic venous pressure gradient.

Peripheral neuropathy is usually the main clinical manifestation of POEMS syndrome and is one of the necessary diagnostic criteria. POEMS syndrome can be differentiated from chronic inflammatory demyelinating polyneuropathy and amyloid degeneration peripheral neuropathy by abnormal M protein levels, elevated vascular endothelial growth factor levels, bone radiation examination, bone marrow biopsy, and skin changes (27). For this patient, POEMS syndrome needed to be differentiated from from hepato-encephalomyelopathy (28, 29); the latter condition mainly manifests as increased muscle tone, progressive spasmodic muscle rigidity, elevated blood ammonia levels, and abnormal directional force and calculation. Monoclonal plasma cell disease is another necessary diagnostic criterion. Monoclonal proteins are detected in the serum and/or urine of approximately 88% of patients, and approximately 40–65% of patients have monoclonal IgA proteins, with almost all light chain types being of the λ type (30). Osteosclerotic lesions most commonly affect the pelvis, spine, ribs, and proximal limbs and must be differentiated from multiple myeloma. The former presents as painless osteosclerotic lesions (unless there are osteolytic lesions), while the latter often presents as painful osteolytic lesions. Elevated serum or plasma VEGF levels are also among the main diagnostic criteria and can be used to evaluate therapeutic effects. Approximately 15% of patients with POEMS syndrome also have lymph node hyperplasia, which is another major criterion. However, because many patients do not undergo lymph node biopsy, these data may be underestimated (17).

The secondary criteria and their estimated incidence rates at diagnosis are as follows: endocrine abnormalities (67%), skin changes (68%), organ enlargement (50%), extravascular volume overload (29%), thrombocytosis/erythrocytosis (50%), and optic disk oedema (29%) (17). The patient had hypothyroidism and hyperprolactinemia, but his blood glucose and parathyroid hormone levels were within normal ranges. Regarding skin changes, the patient had pigmentation on both feet, which needed to be distinguished from pigmentation in patients with liver cirrhosis. The incidence rates of liver enlargement, spleen enlargement, and lymph node enlargement are 68–78%, 35–52%, and 52–61%, respectively (31). For extravascular volume overload, 24% of patients had peripheral oedema. Ascites (7%) and hydrothorax (3%) were less common. A small number of patients may also present with renal involvement. The patient had abnormal renal function, but no pathological biopsy was performed, indicating a possible association with POEMS syndrome. Patients may also develop thrombotic diseases, with decreased protein C and S activity and a tendency toward thrombosis. In addition, lung involvement, such as pulmonary arterial hypertension and pleural effusion, may be observed, but these conditions are not included in the secondary criteria. Survival rates are not affected by the number of characteristics of POEMS syndrome (31, 32).

Effective treatment for POEMS syndrome, such as anti-plasma cell therapy and multisystem adjuvant therapy, are currently lacking. Generally, isolated lesions require surgery or local radiotherapy, while diffuse lesions require systemic treatment. These systemic treatments include chemotherapy regimens such as autologous hematopoietic stem cell transplantation, and lenalidomide combined with dexamethasone (33). If left untreated, progressive peripheral neuropathy may occur, leading to long-term bed rest and high mortality due to cardiovascular dysfunction and infections. The therapeutic effects can be evaluated by assessing VEGF levels, hematological indicators, imaging findings, and symptoms. Studies have also indicated that (34), age > 50 years, hydrothorax, pulmonary arterial hypertension, and epidermal growth factor receptor <30 mL/min/1.73 m^2^ predict poor patient prognosis.

POEMS syndrome has a poor prognosis, with an average median survival period of 5–7 years. Survival depends on the nature and condition of the accompanying disease, and early diagnosis improves patient prognosis. POEMS syndrome has a chronic course, and most patients present with peripheral neuropathy as the first symptom when they seek medical attention (35). Cases studies of POEMS in patients with portal hypertension are limited (36). We summarized the retrieved literature on portal hypertension and POEMS syndrome published in English (Table 2).

**Table 2:** Reports on POEMS and portal hypertension.

Author/Year	Patient	Portal hypertension	Liver biopsy	Clinical outcome
Rie Inoue /2010 (24)	38 years old/ Female	Gastrointestinal bleeding	Dense portal fibrosis and obliteration of small portal vein branches, which are characteristic histological findings of idiopathic portal hypertension (IPH)	The patient died of hepatic encephalopathy at 58 years of age
Debabrata Bandyopadhyay /2013 (20)	42 years old/ Female	Ascites	Mild central lobular scar without bridging fibrosis or nodular regenerative hyperplasia	Symptom relief after oral prednisone, followed by participation in immunomodulator treatment
Sara Campos/ 2017 (37)	48 years old/ Female	Upper gastrointestinal bleeding	Hepatoportal sclerosis, compatible with idiopathic portal hypertension (IPH)	Under a band ligation program, with beta-blocker, diuretics and prophylactic anticoagulation, the patient remains stable
Cyriac Abby Philips/ 2017 (38)	44 years old/ Male	Refractory ascites	Mild portal fibrosis, portal vein dilation, plasma rich portal complications, mild hepatocellular cholestasis, and increased reticuloprotein in the absence of cirrhosis. (idiopathic noncirrhotic portal hypertension, INCPH)	Treatment with nadalidomide, cyclophosphamide, and dexamethasone, and TIPS treatment was recommended
Lina Wu/2017 (39)	46 years old/Male	Ascites	Partial liver cell swelling, occasional punctate necrosis, small amount of lymphocyte infiltration in the portal area, no signs of cirrhosis	After 2 years of treatment with lenalidomide combined with dexamethasone, the patient’s condition improved significantly
Y Chen/ 2022 (36)	62 years old/Male	Oesophageavarices, congested and edematous stomach body, splenomegaly, and transudate ascites	Partial liver cell swelling, visible inclusion bodies in the nucleus, fibrous tissue proliferation in the portal area, lymphocyte infiltration, and small bile duct hyperplasia (IPH)	After TIPS and venous embolization, bleeding symptoms were controlled, and the patient subsequently developed hepatic encephalopathy, ultimately leading to death
Marco Ferronato/2023 (40)	59 years old/Male	Refractory ascites, pleural effusion, and high-risk varices	Irregular distribution of the portal tracts and central veins, consistent with a nonspecific sign of Porto-Sinusoidal Vascular Disorder (PVSD)	Treated with dexamethasone and lenalidomide
Liling Lai/2023 (41)	44 years old/ Male	Ascites	Focal chronic inflammation and bridging fibrosis, with unremarkable staining for myloidosis	After 1 year of treatment with lenalidomide and dexamethasone, the patient’s condition remained stable
Fatima Belabbes/2022 (42)	46 years old/ Male	Dilatation of the splenic and portal vein	Hepatoportal sclerosis, compatible with IPH	Six courses of cyclophosphamide, thalidomide, and dexamethasone were administered, followed by autologous stem cell transplantation to alleviate postoperative symptoms in patients

IPH, idiopathic portal hypertension; INCPH, idiopathic noncirrhotic portal hypertension; PVSD, porto-sinusoidal vascular disorder. Most patients have typical clinical manifestations of POEMS accompanied by portal hypertension, but ascites as the main clinical manifestation is relatively rare.

The diagnostic process of this case was somewhat convoluted. The patient did not have typical clinical signs of liver disease except for ascites. Routine blood routine, liver function tests, coagulation tests, abdominal CT, and liver stiffness tests all supported the initial diagnosis of liver cirrhosis. However, the cause was unknown and common liver diseases related to viral infection, fatty liver, and autoimmune diseases were ruled out. TIPS reduced portal hypertension. Transjugular liver biopsy confirmed the diagnosis as noncirrhotic portal hypertension. However, the symptoms did not improve significantly after the decrease in portal vein pressure. Moreover, the patient’s lower limb weakness gradually increased after TIPS placement, which was not related to postoperative hepatic encephalopathy. After tracing the medical history and performing relevant examinations, the diagnosis of POEMS with portal hypertension was confirmed. The time from onset to diagnosis was 13 months. Because the patient had atypical symptoms, diagnosis was more difficult. Furthermore, the patient was elderly, the condition was severe, and suffered from a concurrent infection after chemotherapy.

This patient had relatively complete diagnosis, treatment, and follow-up data. In the future cardiogenic, nephrogenic, hepatogenic, endocrine, tumor, hematological, and rheumatic immune system diseases should be differentiated in patient presenting with concurrent abdominal fluid, pleural fluid, and a small amount of pericardial fluid. Moreover, some symptoms were overlooked in the patient’s diagnosis and treatment process, including the fact that the patient had symptoms of lower limb weakness 1 year prior and did not undergo thyroid function testing at the first visit. The IgA level was slightly elevated but the globulin level was within the normal range. Further immune electrophoresis and other tests were not performed. The patient’s abdominal CT showed lesions in the lumbar vertebrae, which were not further clarified. Additionally, the serum-pleural effusion albumin gap before and after TIPS was differed (13.1 g/L vs. 6.4 g/L). A decrease in the serum-pleural effusion albumin gap of less than 11 g/L after TIPS was another indication of an etiology other than portal hypertension. Therefore, for noncirrhotic patients with portal hypertension, additional causes, such as rheumatism and blood tumors, should be explored.

## 4 Conclusion

The number of reports on POEMS syndrome has increased in recent years, and POEMS syndrome had gained recognition among clinical physicians. POEMS syndrome is not common and has diverse symptomatic manifestations. Patients with recurrent hydrothorax and ascites as the main manifestations are even rarer (43). To prevent misdiagnosis and missed diagnoses in clinical practice, as well as treatment delays and serious consequences, clinicians should be mindful of this disease.

## Data availability statement

The raw data supporting the conclusions of this article will be made available by the authors, without undue reservation.

## Ethics statement

The studies involving humans were approved by Ethics Committee(seal) of Beijing Youan Hospital Capital Medical University. The studies were conducted in accordance with the local legislation and institutional requirements. The participants provided their written informed consent to participate in this study. Written informed consent was obtained from the individual(s) for the publication of any potentially identifiable images or data included in this article.

## Author contributions

XX: Writing – original draft. CJ: Writing – original draft. TZ: Writing – original draft. MJ: Writing – original draft. YF: Writing – original draft. JL: Writing – review & editing. QM: Writing – review & editing. FX: Writing – review & editing.
